# The Inhibition and Resistance Mechanisms of Actinonin, Isolated from Marine *Streptomyces* sp. NHF165, against *Vibrio anguillarum*

**DOI:** 10.3389/fmicb.2016.01467

**Published:** 2016-09-13

**Authors:** Na Yang, Chaomin Sun

**Affiliations:** ^1^Key Laboratory of Experimental Marine Biology, Institute of Oceanology, Chinese Academy of SciencesQingdao, China; ^2^Laboratory for Marine Biology and Biotechnology, Qingdao National Laboratory for Marine Science and TechnologyQingdao, China

**Keywords:** peptide deformylase, high-throughput screening assay, *Vibrio anguillarum*, marine *Streptomyces* sp. NHF165, resistance mechanism, actinonin

## Abstract

*Vibrio* sp. is the most serious pathogen in marine aquaculture, and the development of anti-*Vibrio* agents is urgently needed. However, it is extreme lack of high-throughput screening (HTS) model for searching anti-*Vibrio* compounds. Here, we established a protein-based HTS screening model to identify agents targeting peptide deformylase (PDF) of *Vibrio anguillarum*. To find potential anti-*Vibrio* compounds, crude extracts derived from marine actinomycetes were applied for screening with this model. Notably, crude extract of strain *Streptomyces* sp. NHF165 inhibited dramatically both on *V. anguillarum* PDF (VaPDF) activity and *V. anguillarum* cell growth. And actinonin was further identified as the functional component. Anti-VaPDF and anti-*V. anguillarum* activities of actinonin were dose-dependent, and the IC_50_ values were 6.94 and 2.85 μM, respectively. To understand the resistance of *V. anguillarum* against actinonin, spontaneous *V. anguillarum* mutants with resistance against actinonin were isolated. Surprisingly, for the resistant strains, the region between 774 and 852 base pairs was found to be absent in the gene *folD* which produces 10-formyl-tetrahydrofolate, a donor of *N*-formyl to Met-tRNA^fmet^. When compared to the wild type strain, Δ*folD* mutant showed eight times of minimum inhibition concentration on actinonin, however, the *folD* complementary strain could not grow on the medium supplemented with actinonin, which suggested that *folD* gene mutation was mainly responsible for the actinonin resistance. To our knowledge, this is the first report showing that marine derived *Streptomyces* sp. could produce actinonin with anti-VaPDF activity and the resistance against actinonin by *V. anguillarum* is mediated by mutation in *folD* gene.

## Introduction

Sudden outbreak of diseases is a major setback in aquaculture, and it leads to high mortality and severe economic loss in all producing countries. Marine *Vibrio* species are associated with large-scale losses of penaeids and also cause diseases to fish (Letchumanan et al., 2015b). *Vibrio anguillarum* is the causative agent of vibriosis, a deadly haemorrhagic septicaemic disease affecting various marine and fresh/brackish water fish, bivalves and crustaceans. In both aquaculture and larviculture, this disease is responsible for severe economic losses worldwide (Frans et al., 2011). *Vibrio* species inhabit aquatic environments at temperatures ranging from 10 to 30°C and are highly susceptible to antibiotics (Shaw et al., 2014). Therefore, antibiotics is one of the main choices for controlling the proliferation of *Vibrio* sp. in aquaculture. Oxytetracycline, tetracycline, quinolones, sulphonamides and trimethoprim are antimicrobial agents permitted and utilized in the Asian aquaculture industry (Letchumanan et al., 2015a). However, extensive use of antibiotics has been postulated to be a major contributing factor in the rising incidence of antimicrobial resistance in pathogenic bacteria. Three fundamental mechanisms of antimicrobial resistance have been summarized: (1) prevention of access to target, (2) changes in antibiotic targets by mutation, and (3) modification (and protection) of targets (Blair et al., 2015). New resistance mechanisms are constantly being described, such as combined novel gene *mph*(G) coding macrolide phosphotransferase and gene *mef*(C) coding eﬄux pump were found to be responsible for high-level macrolide resistance *Vibrio* sp. (Nonaka et al., 2015).

To find novel anti-*Vibrio* sp. agents, screening models targeting *Vibrio* sp. whole cells or proteins involved in quorum sensing have been widely used (Zhang et al., 2016; Zhao et al., 2016). Because of serious antibiotics resistance, screening models with new targets are always needed. Peptide deformylase (PDF) is a class of metalloprotease responsible for catalyzing the removal of *N*-formyl group from *N*-terminal methionine following translation in prokaryotes. The widespread occurrence, conservation, and essential nature of deformylase in bacteria make it an attractive target for antibacterial drug discovery (Giglione et al., 2000; Sangshetti et al., 2015). PDF is widely used in human bacteria infection treatment caused by *Staphylococcus aureus, Streptococcus pneumonia, Helicobacter pylori*, *Haemophilus influenza* and *Mycobacterium tuberculosis*, etc (Sharma et al., 2009; Peyrusson et al., 2015). PDF inhibitors, GSK-1322322, BB-83698 and LBM-415, have entered into clinical developments (Sangshetti et al., 2015).

However, very little was investigated about PDF of aquaculture pathogen *V. anguillarum*. Actually, like other gram-negative organisms, *V. anguillarum* has one chromosomal copy of *pdf* gene, and no results have been published regarding PDF as an anti-*Vibrio* sp. target in marine aquaculture. Actinonin was reported in 1962 (Gordon et al., 1962) and was the first characterized PDF inhibitor (Chen et al., 2000). Up to now, resistance to actinonin has been reported in *Staphylococcus aureus, Streptococcus pneumonia, Bacillus subtilis, Haemophilus influenza, Streptococcus pyogenes* and *Escherichia coli*. Mechanisms causing actinonin resistance were also investigated in these strains. Genes *pdf*, *folD*, *fmt*, and *glyA* involved in translation initiation were the most frequency mutation sites (Margolis et al., 2000, 2001; Duroc et al., 2009).

Natural products are essential for the novel antibiotics screening. A lot of compounds had been developed to efficient antibiotics and applied in diseases treatment of human and aquaculture (Varoglu et al., 1997; Vinothkumar and Parameswaran, 2013). It is well known that the biodiversity of the marine environment and the associated chemical diversity constitute a practically unlimited resource of new bioactive substance, and the bioactive compounds from marine microorganisms have been exploited for decades (Varoglu et al., 1997). Marine actinomycete is one of the most efficient organisms of natural bioactive metabolite producers. The genus *Streptomyces* is considered as the most prolific producer of bioactive agents amongst actinomycete (Miao and Davies, 2010). Interestingly, *Streptomyces* sp. isolated from arctic were found to have biofilm inhibitory activity against *Vibrio* sp. by attenuating the signal molecules *N*-acylated homoserine lactones’ activity (You et al., 2007), and *Streptomyces* producing siderophores derived from nearshore marine sediments were found to inhibit the growth of *Vibrio* sp. by competition for iron in the aquatic environment (You et al., 2005).

In this study, we established an high-throughput screening (HTS) model targeting PDF of pathogenic bacterium *V. anguillarum* YN isolated from infected *Scophthalmus maximus* samples. Actinomycetes from eight different South China Sea sediments were isolated and corresponding crude extracts were prepared and subjected to anti-*V. anguillarum* agents screening. Actinonin produced by marine *Streptomyces* sp. NHF165 exhibited high inhibitory both on *V. anguillarum* PDF (VaPDF) activity and *V. anguillarum* cell growth. Furthermore, actinonin-resistant *V. anguillarum* mutants were obtained and the mechanism of resistance was also elucidated.

## Materials and Methods

### *V. anguillarum* PDF (VaPDF) Expression and Purification

The *pdf* gene was amplified from *V. anguillarum* YN genome DNA by PCR using the following primers: For: 5′-CGCGGATCCATGTCTGTATTACAAG-3′ (the underlined region indicates *Bam*H I site) and Rev: 5′-CCGCTCGAGTTAGTTTTTTTCGTTATAG-3′ (the underlined region indicates *Xho* I site). PCR products were cloned into pMD18-T vector (TaKaRa). After sequence confirmation, PCR products were inserted in the multiple cloning site of vector pET30a(+) (Novagen) and the resulting plasmid was designated as pET30a(+)::*pdf*. Plasmid pET30a(+)::*pdf* was transformed into *E. coli* BL21(DE3) cells. Recombinant PDF was expressed and purified as follows. Briefly, cells harboring plasmids pET30a(+)::*pdf* were grown to an absorbance at 600 nm (*A*_600_) of 0.6 and induced with 0.5 mM isopropyl-D-thiogalactopyranoside at 16°C overnight. Cells were harvested by centrifugation, washed in HEPES buffer (25 mM, pH 7.4) and resuspended in HEPES (pH 7.4)-75 mM KCl-10% glycerol (buffer A). Then cells were lysed by sonication and centrifugated at 25,000 ×*g*. The supernatant was loaded onto a 5 ml HisTrap FF column (GE healthcare) and equilibrated in buffer A. The column was further washed and eluted with a gradient of imidazole from 0 to 300 mM using ÄKTA protein purification system (GE healthcare).

### Anti-VaPDF Screening Assay

Peptide deformylase catalyzes the removal of the *N*-formyl group from formyl-Met-Ala-Ser. The free amino group reacts with fluorescamine to form highly fluorescent products which can be monitored with a TECAN Infinite M1000 PRO multi-mode microplate reader by exciting at 390 nm and emission at 470 nm. For screening, assays were performed in black flat-bottom 96-well microplates (Corning). First, 49.5 μl reaction solution (20 nM VaPDF, 1 mM formyl-Met-Ala-Ser and 25 mM HEPES, pH 7.4) was dispensed in each well and then 0.5 μl dimethylsulfoxide (DMSO) or samples dissolved in DMSO (4 mg/ml) was dispensed. Plates were incubated at 37°C for 30 min. Then fluorescamine was added to a final concentration of 60 μg/ml. The fluorescence intensity (FI) of each well was detected. The inhibitory values were calculated as (FI_sample_-FI_negativecontrol_)/(FI_positivecontrol_-FI_negativecontrol_) × 100%.

Dimethylsulfoxide was chosen as negative control and heat-inactivated VaPDF as positive control during measurements. The *Z*′ factor and CV values were calculated as follows:

*Z*′ = 1–3(SD_FImax_-SD_FImin_)/(Mean_FImax_-Mean_FImin_), SD: standard deviation. The theoretical value is between 0.5 and 1. CV(%) = SD_FImax_/Mean_FImax_ or CV(%) = SD_FImin_/Mean_FImin_. The acceptable value of CV for HTS assay is less than 10%.

### Anti-*V. anguillarum* Cell Based Assay

The anti-*V. anguillarum* assay utilized strain *V. anguillarum* YN which was isolated from infected *Scophthatmus maximus* sample. The activities of crude extracts or compounds against *V. anguillarum* were determined in a clear flat-bottom 96-well plate. *V. anguillarum* YN was grown at 28°C to mid-log phase in Luria Bertani (LB) medium ( peptone 10 g, yeast extract 5 g, NaCl 10 g, in 1000 ml distilled water, pH 7.0). Then the culture was diluted to *A*_600_ = 0.025 with LB medium. 80 μl bacterial suspension was added to each well, followed by adding 0.8 μl of sample solution (4 mg/ml). DMSO served as the negative control and chloramphenicol as the positive control. The plate was incubated at 28°C for 15 h and the growth of *V. anguillarum* YN was measured by detecting *A*_600_ of each well.

### Marine Actinomycetes Isolation and Crude Extracts Preparation

Sediment samples were collected using the mud sampler in the South China Sea during 26th April to 23th May 2010 (Supplementary Table S1). The samples were transported to laboratory in an insulated container at 4°C and then stored at -80°C. All samples were pretreated using dispersion and differential centrifugation (DDC) method (Hopkins et al., 1991) to enrich for spore-forming actinomycetes. Five different agar media were selected for spreading sediment samples: (1) M1 agar: raffinose 10.0 g, L-histidine 1.0 g, K_2_HPO_4_ 1.0 g, MgSO_4_.7H_2_O 0.5 g, FeSO_4_.7H_2_O 0.01 g, agar 15.0 g; (2) M2 agar: trehalose 5.0 g, proline1.0 g, (NH_4_)_2_SO_4_ 1.0 g, NaCl 1.0 g, CaCl_2_ 2.0 g, K_2_HPO_4_ 1.0 g, MgSO_4_.7H_2_O 1.0 g, agar 20.0 g; (3) M3 agar: humic acid 1.0 g, KCl 1.7 g, NaH_2_PO_4_ 0.5 g, MgSO_4_.7H_2_O 0.5 g, FeSO_4_.7H_2_O 0.01 g, CaCO_3_ 0.02 g, agar 15.0 g; (4) M4 agar: glycerol 12.5 g, arginine 1.0 g, K_2_PO_4_ 1.0 g, NaCl 0.5 g, MgSO_4_.7H_2_O 0.5 g, CuSO_4_.5H_2_O 0.001 g, trace salt solution 1.0 ml, agar 15.0 g, trace salt solution contains FeSO_4_.7H_2_O 0.001 g, MgCl_2_.4H_2_O 0.001 g, ZnSO_4_.7H_2_O 0.001 g, distilled water 1000 ml; (5) M5 agar: soluble starch 10.0 g, hydrolyzed casein 0.3 g, NaCl 5.0 g, KNO_3_ 2.0 g, K_2_HPO_4_ 2.0 g, MgSO_4_.7H_2_O 0.5 g, CaCO_3_ 0.02 g, FeSO_4_.7H_2_O 0.01 g, agar 15.0 g. All media were prepared using the artificial seawater and adjusted to pH 7.5 and were supplemented with nalidixic acid (20 μg/ml) and nystatin (100 μg/ml) or cycloheximide (100 μg/ml) to inhibit the growth of fungi and Gram-negative bacteria. Spreaded plates were incubated at 28°C for 1 month. Actinomycetes were selected and transferred to GT agar medium until pure cultures were obtained for further study (GT agar medium: soluble starch 20 g, L-asparagine 0.5 g, KNO_3_ 1.0 g, K_2_HPO_4_.H_2_O 0.5 g, NaCl 0.5 g, MgSO_4_.7H_2_O 0.5 g, distilled water 1000 ml, pH 7.5). Pure actinomycetes were maintained on GT slants at 4°C and 25% (v/v) glycerol suspensions at -80°C. Morphological features of spores and mycelia were observed by light microscopy (model BH2; Olympus) and scanning electron microscopy (Quanta 200). For crude extracts preparation, all the selected strains were cultured in 250 ml flask containing 40 ml fermentation medium (MPG medium consisting of glucose 10.0 g, millet meal 20.0 g, cotton seed gluten meal 20.0 g, MOPS 20.0 g, distilled water 1000 ml, pH 7.2). The liquid cultures were grown for 7 days at 28°C with shaking at 160 rpm. An equal volume of ethyl acetate was added to the liquid cultures for extraction and evaporated to give crude extracts.

### 16S rRNA Gene Amplification and Phylogenetic Analysis

The 16S rRNA genes were amplified by using universal bacterial primers: 27F and 1492R (Lane, 1991). PCR products were sent to Sangon Biotech (Shanghai, China) Co. Ltd. for DNA sequencing and deposited in GenBank (accession numbers: KU500358- KU500370, KU312336- KU312339, KU529470- KU529472, KU550963, JQ911670). The 16S rRNA gene sequences were compared with available 16S rRNA gene sequences from GenBank database by using BLAST program^1^ to determine an approximate phylogenetic affiliation. Neighbour-joining (NJ) tree was constructed using software package Mega version 6.0 (Tamura et al., 2013). Bootstrap re-sampling method with 1000 replicates was used in evaluating the topology of the phylogenetic trees (Felsenstein, 1985).

### Compound Separation and Identification

The fermentation of active strain *Streptomyces* sp. NHF165 was carried out in 1000 ml flask containing 250 ml MPG medium that inoculated 3 ml seed culture of strain *Streptomyces* sp. NHF165. The fermentation broth was cultured at 28°C for 7 days on a rotary shaker at 160 rpm. After fermentation, total broth (10 L) was fractionated by centrifugation. Supernatant was extracted with the same volume ethyl acetate thrice. The evaporated ethyl acetate phase crude extract was applied on a Sephadex LH-20 column [elution reagent, dichloromethane:methanol = 2:1 (v/v)] and separated into 10 fractions. The sixth fraction with anti-VaPDF activity was subjected to a preparative HPLC C18 column (9.4 mm × 250 mm, 5 μm, Agilent) using acetonitrile and water as mobile phase at 3 ml/min to give pure compound 1 (5.2 mg) and 2 (3.5 mg). And the compounds were identified by checking NMR data.

### Resistant Mechanism Study of Actinonin against *V. anguillarum*

To isolate *V. anguillarum* resistant to actinonin, exponential-phase cells were inoculated into Mueller-Hinton (MH) broth supplemented with 25 μM of actinonin and incubated for 1 day at 28°C. Then 100 μl culture was plated onto MH agar containing 25 μM of actinonin. Resistant colonies were picked and restreaked for single-cell colonies on the same plate. Purified resistant mutants were frozen at -80°C in LB with 10% DMSO. Growth curves for wild type and mutant strains were tested using MH broth without actinonin at 28°C for 25 h. The growth was monitored at different time points by reading *A*_600_. Cells were also plated on minimal medium (MM) agar (Duroc et al., 2009) to test the growth. For MICs (minimum inhibition concentration) determination, actinonin was serially diluted twofold from 1000 to 0.49 μM in each column using a clear flat-bottom 96-well plate. The plate was incubated at 28°C for 15 h, and after incubation, the plate was read under absorbance at 600 nm. In this study, the MIC was defined as the lowest actinonin concentration which prevented *V. anguillarum* growth (an *A*_600_ value < 0.05).

The PCR primers used for DNA amplification of the *pdf*, *folD*, *fmt*, and *glyA* genes were designed from the appropriate sequences of the corresponding public genome sequences from NCBI website^2^. PCR amplification was performed with both wild type and mutants genome DNAs of *V. anguillarum*. PCR products were confirmed by sequencing in Sangon Biotech (Shanghai, China) Co. Ltd. Alignment of the DNA sequences of the *pdf*, *folD*, *fmt*, and *glyA* genes from wild type and mutant strains was carried out using software package Mega version 6.0. To confirm whether mutation of gene *folD* leads to resistance, complementary experiment was taken out. Briefly, full length of *folD* was amplified from wild *V. anguillarum* genome DNA by PCR and ligated into vector pACYC184 (Milton et al., 1992), which was transformed conjugately into mutant *V. anguillarum* by a donor strain *E. coli* 17-1. The positive clones were selected on LB agar containing tetracycline.

Expression changes in transcription level between wild type and Δ*folD* strain were compared by performing RT-PCR. RNA was extracted from 2 ml culture broth of bacterial samples using an Ultrapure RNA Kit (CWBio) as described by the manufacturer. 1 μg total RNA of each sample was subjected to reverse transcription using random hexamers to prepare cDNAs. RT-PCR was optimized with a SYBR Premix Ex Taq kit (TaKaRa) for each primer pair (**Table 1**). Each cDNA sample was independently quantified three times, with two technical replicates of each. Relative mRNA levels were calculated.

**Table 1:** Activity assays of marine actinomycetes crude extracts.

Strain number	Sediment sample number	Closest species	Anti-*V. anguillarum* activity (%)	Anti-PDF activity (%)
NHF7	54	*Streptomyces labedae*	39.2 ± 3.4	0
NHF15	54	*Nocardiopsis lucentensis*	18.1 ± 5.0	25.3 ± 11.3
NHF22	54	*Nocardiopsis lucentensis*	9.8 ± 6.3	28.3 ± 5.0
NHF26	54	*Nocardiopsis valliformis*	3.8 ± 3.2	28.8 ± 2.2
NHF27	76	*Nocardiopsis lucentensis*	20.8 ± 3.6	37.7 ± 4.7
NHF28	33	*Prauserella marina*	34.9 ± 2.7	0
NHF45	37	*Salinispora arenicola*	0	0
NHF48	31	*Nocardiopsis lucentensis*	12.9 ± 3.1	42.1 ± 0.9
NHF57	37	*Micromonospora humi*	10.0 ± 6.0	49.9 ± 0.12
NHF61	37	*Promicromonospora aerolata*	0	0
NHF69	76	*Micromonospora aurantiaca*	0	0
NHF86	33	*Streptomyces violascens*	18.0 ± 3.4	50.0 ± 2.5
NHF90	65	*Streptomyces praecox*	21.5 ± 3.8	0
NHF93	32	*Streptomyces griseoplanus*	4.9 ± 1.6	0
NHF97	31	*Streptomyces anulatus*	37.9 ± 5.3	22.2 ± 10.0
NHF107	54	*Prauserella marina*	0	0
NHF129	37	*Micromonospora* sp.	42.5 ± 2.7	0
NHF132	65	*Saccharopolyspora spinosa*	0	0
NHF133-2	65	*Saccharopolyspora spinosa*	0	0
NHF142-1	69	*Verrucosispora gifhornensis*	0	0
NHF148	69	*Micromonospora carbonacea*	0	0
NHF165	32	*Streptomyces cacaoi* subsp. *cacaoi*	70.0 ± 8.3	49.1 ± 20.6

## Results

### Establishment and Validation of Screening Model Targeting VaPDF

**FIGURE 1:**
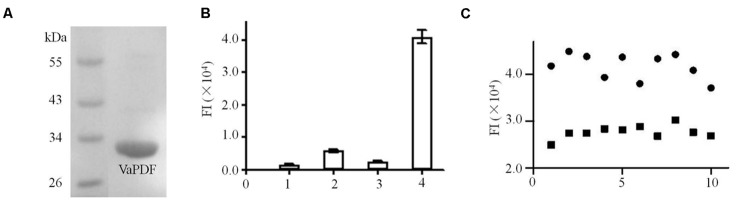
**Purification and activity assays of *Vibrio anguillarum* PDF (VaPDF). (A)** The purified VaPDF with 6 × His tag. **(B)** Activity assays of purified VaPDF. 1, HEPES buffer. 2, HEPES buffer + formyl-Met-Ala-Ser. 3, HEPES buffer + VaPDF. 4, HEPES buffer + formyl-Met-Ala-Ser + VaPDF. Data are representative of three independent experiments. Error bars indicate standard deviation. **(C)** Validation of VaPDF assay. Fluroscence intensity of positive controls and negative controls were detected (*n* = 10). Solid circle, negative control; solid square, positive control.

The genome sequence of *V. anguillarum* on NCBI web was used as a major reference to clone the *pdf* gene. The sequencing result showed that the length of *pdf* gene of *V. anguillarum* YN was 510 bp (including stop codon) which encodes a 19.21 kDa “Class I” PDF (Giglione et al., 2000) (**Figure 1A**), and the GenBank accession number of this gene is KU214433. BLAST result showed that its encoding protein VaPDF had 98.0% identity to other types of *Vibrio* sp. PDFs in amino acid sequence. VaPDF shared three highly conserved characteristic stretches (Baldwin et al., 2002): motif 1 (GIGLAATQ), motif 2 (EGCLS), and motif 3 (HELDH) (Supplementary Figure S1) with other types of PDFs.

Activity of targeting protein is essential for the establishment of screening model. Based on previous data, PDFs purified from *Leptospira interrogans* etc. catalyzed the removal of a formyl group from the N-termini of nascent polypeptides (Li et al., 2002). Consistently, the purified VaPDF catalyzed the removal of the *N*-formyl group from formyl-Met-Ala-Ser (**Figure 1B**) and the free *N*-formyl group could reacted with fluorescamine to form highly fluorescent products. The optimized reaction conditions were determined as 40 nM VaPDF, 1 mM substrate in 25 mM HEPES buffer (pH 7.4) for 30 min at 37°C. The VaPDF screening model can tolerate up to 2% DMSO (Supplementary Figure S2). Moreover, the Z′ factor was calculated in order to evaluate the PDF assay for HTS. In this model, the value of Z′ factor was 0.71(≥0.5) which is considered acceptable for HTS. The CV values were CV_FImax_ = 6.7% and CV_FImin_ = 5.1%. Both values were less than the threshold value of 10% that is recognized as delineation of correct assays (**Figure 1C**).

### Selective Isolation of Actinomycetes

**FIGURE 2:**
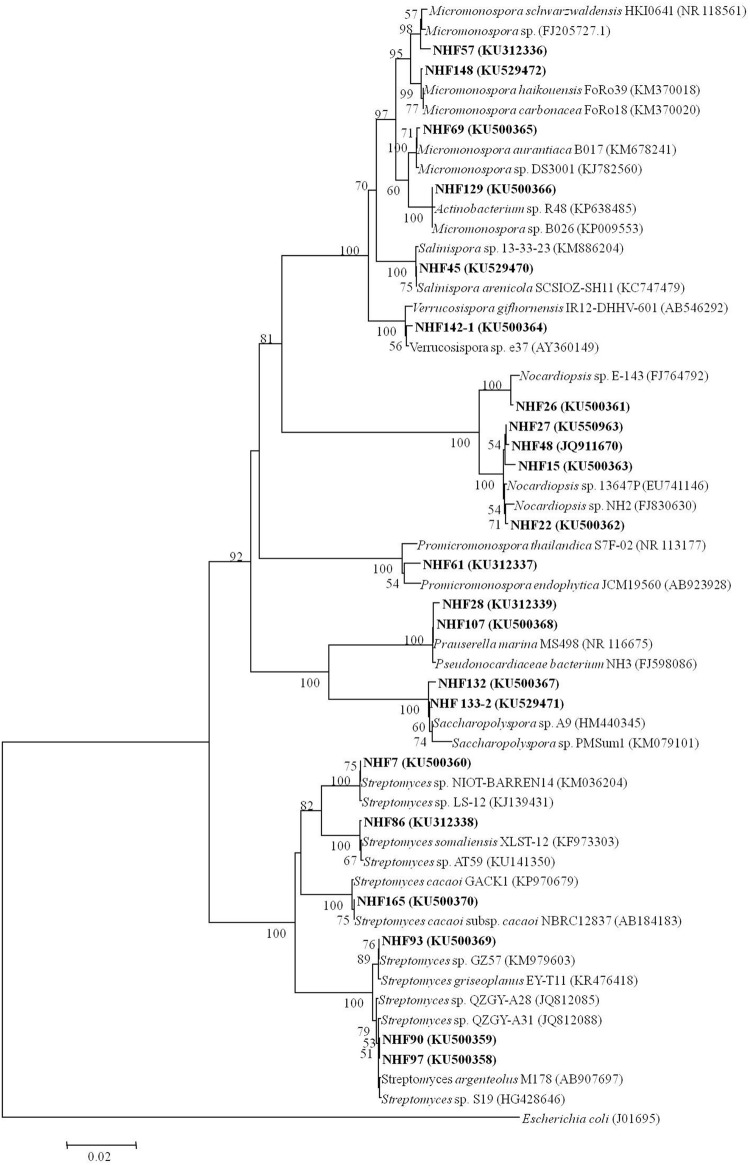
**Neighbor-joining tree showing the phylogenetic relationships of actinobacterial 16S rRNA gene sequences of obtained strains from South China Sea sediments.** Bar, 0.02. Bootstrap values of >50% (for 1000 replicates) are shown.

To find potential novel compounds against *V. anguillarum* with our HTS model mentioned above, we sought to isolate marine actinomycetes derived natural products for the screening. Totally, 84 actinobacterial strains were isolated from eight marine sediment samples based on the characteristic colonial morphology. As expected, the predominant population of marine actinomycetes was similar to the previous report with marine sediment samples (Maldonado et al., 2005), which showed that *Streptomyces* was the most abundant species, then was the *Micromonospora*. Other rare actinomycetes were also recovered from sediment samples. Thereafter, 22 strains were selected and subjected to 16S rRNA gene sequence analysis. GenBank accession numbers were shown in **Table 1**. Results indicated that these 22 strains shared 99% of similarities with their closest strains. And they belonged to eight genera, which were *Micromonospora, Nocardiopsis*, *Prauserella*, *Promicromonospora, Saccharopolyspora*, *Salinispora, Streptomycetes*, and *Verrucosispora*. The phylogenetic affiliation was investigated and the results were presented in **Figure 2**.

### HTS for Crude Extracts of Marine Actinomycetes

**FIGURE 3:**
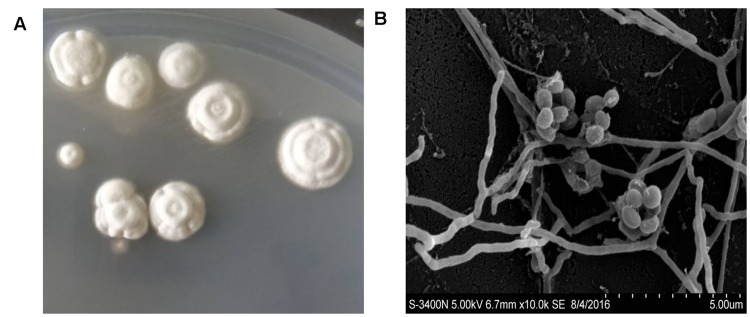
**Characteristics of *Streptomyces* sp. NHF165. (A)** Colony characteristics of *Streptomyces* sp. NHF165. **(B)** Scanning electron micrograph of *Streptomyces* sp. NHF165 grown on GT agar at 28°C for 7 days. Bar = 5 μm.

To identify the anti-VaPDF activity of different marine actinomycetes mentioned above with the present HTS model, the corresponding crude extracts were prepared with ethyl acetate extraction method. Thereafter, the crude extracts were used for screening to discover anti-VaPDF agents. For the first round screening, each crude extract was added to a final concentration of 20 μg/ml to the reaction system. Screening results showed that crude extracts isolated from strains NHF27, NHF48, NHF57, NHF69, NHF86, and NHF165 exhibited anti-VaPDF activity with minimum 30% inhibition. Active crude extracts were produced by strains affiliated to genera *Micromonospora, Nocardiopsis*, and *Streptomyces.* To confirm the anti-vibrio activities of above active crude extracts, anti-*V. anguillarum* YN cell activity results were also checked and shown in **Table 1**. Notably, crude extract isolated from strain *Streptomyces* sp. NHF165 exhibited the highest inhibitory both on VaPDF activity and *V. anguillarum* YN cell growth. Therefore, *Streptomyces* sp. NHF165 was chosen for further study. Strain NHF165 had a highest 16S rRNA gene similarity (>99%) with *Streptomyces cacaoi* subsp. *Cacaoi*, and colonies of this strain appeared to be yellow substrate mycelium and white aerial mycelium. Oval spores were produced along the long, straight and smooth aerial mycelium after 7 days of cultivation on medium GT (**Figure 3**).

### Structure Elucidation of Compounds Produced by *Streptomyces* sp. NHF165

**FIGURE 4:**
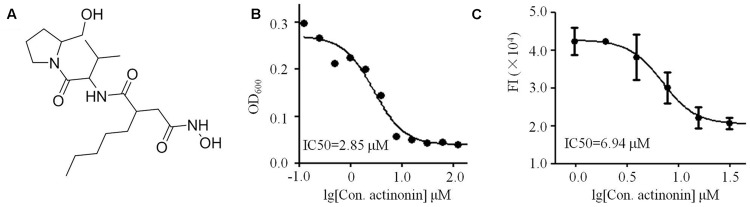
**Characterization of marine derived actinonin. (A)** Structure of actinonin isolated from *Streptomyces* sp. NHF165. **(B)** Anti-*V. anguillarum* IC_50_ value of actinonin. **(C)** Anti-VaPDF IC_50_ value of actinonin.

To identify the exact structure of compound with anti-VaPDF activity isolated form *Streptomyces* sp. NHF165, the corresponding crude extract was separated with sephadex LH-20. The purification results showed that fraction 6 contained the main anti-VaPDF constitute. Then fraction 6 was further separated with HPLC with C18 column and two compounds were finally obtained (**1** and **2**). Their structures were elucidated by UV, 1D NMR, 2D NMR (^1^H-^1^H COSY, ^1^H-^13^C HSQC, ^1^H-^13^C HMBC). ESI-MS data revealed molecular ion peaks at *m/z* 386.2961 [M+H]^+^, and 408.2498 [M+Na]^+^ for compound **1** (Umezawa et al., 1985). The compound **1** with anti-VaPDF activity was identified by comparing the NMR data with previous published data, and it was considered to be actinonin (**Figure 4A**) (Umezawa et al., 1985). The total yield of actinonin was 5.3 mg per 10 L broth. Correspondingly, this marine derived-actinonin inhibited the VaPDF activity in a dose-dependent manner and the IC_50_ was 6.94 μM. The IC_50_ of this actinonin on *V. anguillarum* cell viability was 2.85 μM (**Figures 4B,C**).

Compound **2** was obtained as light brown amorphous powder. Its HRESIMS revealed a molecular ion peak of *m/z* 565.2565 for C_32_H_33_N_6_O_4_ [M+H]^+^ (Calcd. 565.2485) and suggested 564 as the molecular weight and C_32_H_32_N_6_O_4_ as the molecular formula. UV spectrum with the maximal absorbance at 206, 228, and 288 nm.^13^C NMR spectrum of compound **2** revealed signals of 32 carbons, including four amide carbonyl ^13^C resonances were suggested by signals of δ_C_ 165.2, C-13; 165.6, C-35; 166.0, C-16; 169.1, C-32. The ^1^H and ^13^C NMR spectra in combination with ^1^H–^1^H COSY and ^1^H–^1^C HSQC NMR data indicated signals of two substituted benzene groups (1, 2- substituted benzene: δ_H_ 7.16, *d*, 12.0, δ_C_ 123.8, C-5; δ_H_ 6.62, *t*, overlap, δ_C_ 118.1, C-6; δ_H_ 6.98, *t*, 7.2, δ_C_128.1, C-7; δ_H_ 6.65, *d*, 6.0, δ_C_ 109.5, C-8; δ_C_ 133.0, C-4; δ_C_ 149.4, C-9 and 1, 2, 4- substituted benzene: δ_H_ 7.21, d, 6.0, δ_C_ 111.5, C-20; δ_H_ 7.03, *d*, 12.0, δ_C_ 119.2, C-21; δ_H_ 7.62, *s*, δ_C_ 114.6, C-23; δ_C_ 134.1, C-22, δ_C_ 127.1, C-24; δ_C_ 134.8, C-25). ^1^H–^13^C HMBC NMR data revealed HN-26 connected with C-24, C-25, C-27 (δ_C_ 124.7), C-28 (δ_C_ 109.6), and H-27 connected with C-24 and C-25. The 1, 2, 4-substituted benzene moiety was an indole structure. Combined ^13^C and HMBC spectrum, C-30, 32, 33, 35, 36, 37, 38 signals showed a diketopiperazine moiety. H-29 [δ_H_ 3.23, dd (14.4, 4.2); 3.06, dd (12.0, 6.0)] connected with C-24, C-27, C-35, and HN-31 (δ_H_ 7.7) connected with C-32, C-35. These data suggested this group was a condensation product of tryptophan and proline. The HMBC signals from H-2 (δ_H_ 5.63, *s*) to C-4, C-9 and from HN-1 (δ_H_ 6.61) to C-2 (δ_C_ 81.1), C-3 (δ_C_ 58.7), C-4, C-8, and C-9 demonstrated that the 1, 2- substituted benzene moiety was an indoline structure. C-11, 13, 15, 16, 17, 18, 19 signals were assigned to another diketopiperazine moiety. A methylene group contributed to establish connectivity of indoline and diketopiperazine moieties. Signal from H-2 to C-16 demonstrated the connection of C-2 to N-10. Signal from H-2 to C-22 showed the connection of C-3 to C-22. ROESY data showed signals from H2 to H-11 and H-21 which suggested H-1, H-11 and indolyl diketopiperazine structure on the same side. Thus the structure of **2** was established (Supplementary Figure S3). It was apparent that compound **2** was related to asperazine derived from a marine fungi *Aspergillus niger* (Varoglu et al., 1997). Compound **2** was shown to be a new compound of indolyl diketopiperazine analogs, and it showed no activities against *V. anguillarum* or VaPDF.

### Resistance Mechanism of *V. anguillarum* against Actinonin

**FIGURE 5:**
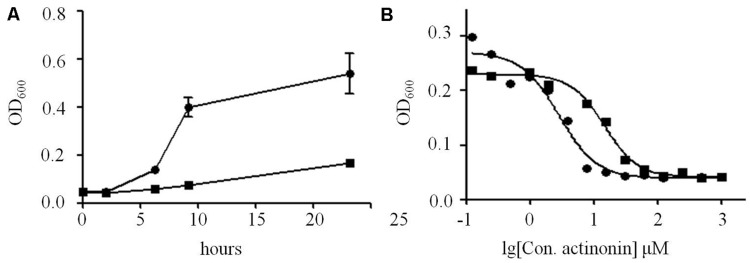
**Proliferation differences between *V. anguillarum* mutant and wild type strains. (A)** Growth of *V. anguillarum* mutants and wild type strains in MH broth, measured as optical density at 600 nm. **(B)** MIC tests of actinonin on *V. anguillarum* mutants and wild type strains. Solid circle, wild type strain; solid square, mutant strain.

The resistance of *V. anguillarum* YN to actinonin was challenged on MH agar with 25 μM actinonin. The frequency of resistance in *V. anguillarum* YN was 5 × 10^-6^. Notably, the mutants were stable, as re-streaking on actinonin-free MH agar did not lose resistance, and no phenotypic differences between wild type and mutant were observed for this strain. Compared with parent strains, *V. anguillarum* YN mutants grew at much slower rates when cultured in MH broth (**Figure 5A**) and showed 8 × MIC to actinonin (**Figure 5B**). Moreover, these mutants showed resistance to actinonin but still remained susceptibility to streptomycin, chloramphenicol, carbenicillin, kanamycin, and ampicillin as wild type strains do.

In order to understand the mutation details, open reading frame regions of *pdf*, *folD*, *fmt*, and *glyA* DNA sequence from the mutant strains were amplified, sequenced and aligned with those from parent strains. The results showed that no mutation was retrieved in *pdf*, *fmt*, and *glyA*, and all five mutant strains harbored a mutation in *folD* gene possessing deletion of base pairs 774–852 (Supplementary Figure S4). As reported, *folD* catalyzes the formation of 10-formyl-tetrahydrofolate (THF), which supplies *N-formyl* group to Met-tRNA^fMet^. To our knowledge, Δ*folD* mutants have been described only in species *Salmonella enterica* and *B. subtilis* (Duroc et al., 2009). None of the resistant strains could grow on MM medium, which consisted with the results described previously (Duroc et al., 2009). To determine whether mutation of gene *folD* is the main cause for the actinonin resistance of *V. anguillarum*, complementary experiment was performed. Plasmid pACYC184::*folD* was successfully constructed and introduced into Δ*folD* mutants to get pACYC184::*folD/*Δ*folD* strains. Complementary strains could not grow on MH agar with 25 μM actinonin in this study, which further confirmed that *folD* gene mutation was responsible for actinonin resistance in *V. anguillarum*.

**FIGURE 6:**
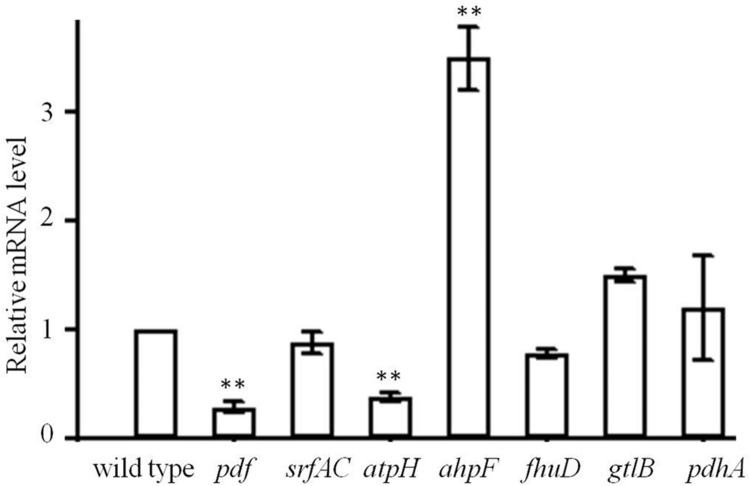
**Trancription level of *pdf, srfAC, atpH, ahpF, fhuD, gtlB*, and *pdhA* in the mutant of *V. anguillarum*.** Wild type was used as a control. Values reported are the mean of three replicates. Error bars indicate the standard deviation. ^∗∗^*p* < 0.01 was taken to indicate statistical distinct significance.

To understand the expression changes between wild type and mutant strains, genes involved in translation initiation (*pdf*), amino acid biosynthesis (*gtlB*), metabolites biosynthesis (*srfAC*), ATP production (*atpH*), cell protection (*ahpF*), ABC transporter (*fhuD*), TCA cycle (*pdhA*) were checked with RT-PCR (Supplementary Table S2) and the expression of *rplL* gene was used as a reference for the determination of induction levels. Significant expression changes of *pdf*, *atpH*, and *ahpF* genes were observed for genes encoding functions of the intermediary metabolism (**Figure 6**). pdf and *atpH* genes were significantly down-regulated, which suggested that the translation initiation was hampered by less *N-formyl* group supply. However, the expression of gene *ahpF* corresponding for protecting cells was significantly up-regulated. Thus, in the tested condition, *V. anguillarum* mutants developed an adaptation mechanism to survive in high concentration of actinonin.

## Discussion

*Vibrio anguillarum* is an opportunistic fish pathogen that is common to marine and estuarine environments. It has been identified as the main cause of vibriosis, a potentially fatal septicemia that affects fish and shellfish in marine aquaculture, with consequent economic losses (Frans et al., 2011). To find novel antibiotics against *V. anguillarum* is urgently needed.

It is now widely accepted that the traditional screening methods are unlikely to generate many promising molecules. Alternative strategies must therefore be developed to find new compounds. One possible strategy is to identify a molecular target at the outset and then to screen the available libraries of chemical compounds looking for hits with potent inhibitory capacities *in vitro* with HTS model. However, it is extreme lack of HTS model for searching anti-*Vibrio* compounds. For this approach, the identification of a good target is vital. PDF has been suggested as a possible candidate that may fulfill all those criteria for HTS and has become a promising and attractive bacterial target to explore for the discovery of new antibacterial agents (Giglione et al., 2000). We confirmed VaPDF shared the three highly conserved characteristic stretches and was essential for *V. anguillarum* growth. Therefore, active agents against VaPDF can be potential drugs for vibriosis treatment. Due to the lack of effective anti-*Vibrio* HTS methods, we first developed a protein-based assay based on VaPDF activity and screened crude extracts derived from marine actinomycetes.

In recent years, great attention has been paid to the isolation and characterization of actinomycetes from marine environment, which provides a valuable source for discovering bioactive metabolites. South China Sea located in the southeast of China with tropical oceanic climate and was poorly studied. Therefore, we chose deep-sea sediment samples collected from South China Sea to isolate anti-*Vibrio* actinomycetes, which might be used in marine aquaculture industry. Totally, 84 actinobacterial strains belonging to eight genera were obtained. The predominant numbers of *Streptomyces* and *Micromonospora* strains is in line with the results reported previously (Maldonado et al., 2005). Representative strains isolated in the present study showed bioactivities against VaPDF and *V. anguillarum* cell. Among 22 strains, 14 strains showed anti-bacteria activity against *V. anguillarum* and 9 strains showed anti-activity against VaPDF. These strains belonged to genera *Streptomyces, Micromonospora*, and *Nocardiopsis*.

As is well known, *Streptomyces* could produce diverse range of secondary metabolites with relevant anti-inflammatory, antimicrobial, antioxidant activities (Dubert et al., 2015) and are potential probiotics in aquaculture (Tan et al., 2016). *Streptomyces rubrolavendulae* M56 isolated from the sediments of Bay of Bengal could significantly exclude the pathogenic *Vibrio* spp. in co-culture experiments (Augustine et al., 2015). Addition of 1% wet cell mass of marine *Streptomyces* strains can reduce mortality rate of nauplii and adult Artemia caused by both *V. harveyi* and *V. proteolyticus* (Das et al., 2010). Crude extract of *Streptomyces* sp. LCJ94 showed good inhibitory activities against *V. harveyi*, *V. vulnificus*, *V. alginolyticus* with the MIC values of 250, 250, and 500 μg/ml, respectively (Mohanraj and Sekar, 2013). In this study, *Streptomyces* sp. NHF165 exhibited the highest activity against *V. anguillarum*, and the functional component was finally determined as actinonin. Actinonin was isolated from soil *Streptomyces* in 1962 and was reported to be an inhibitor targeting *E. coli* PDF and *M. tuberculosis* PDF (Sharma et al., 2009). Our discovery is the first report to show that marine derived actinonin possesses anti-*Vibrio* activity via targeting VaPDF. Considering *Streptomyces* sp. NHF165 with high yield (5.3 mg/10 L) and low IC_50_ of actinonin on *V. anguillarum* (2.85 μM), it might be a good candidate for the management of vibriosis in marine aquaculture industry. On the other hand, as a natural product, actinonin shows derivative of L-prolinol and hydroxamic acid of the type R-CO-NHOH and some structural relationship to other polypeptide antibiotics. Hence, it will be very interesting to dig the conserved DNA sequence of non-ribosomal peptide synthetases (NRPS) adenylation domain (Ayuso-Sacido and Genilloud, 2005) in the genomic DNA of *Streptomyces* sp. NHF165 in the future.

Nowadays, antibiotics have been routinely applied to water to treat and prevent bacterial disease in fish and shellfish culture industries. However, extensive use of antibiotics goes with development of resistant strains, especially resistant vibrios. Characterization of antibiotic-resistant vibrios is necessary to elucidate mechanism of resistance. *Vibrio* strains with resistance to chloramphenicol, tetracycline, amoxicillin, or streptomycin were successfully isolated from hatchery larval cultures, and R-plasmids harboring resistant genes (chloramphenicol acetyltransferase, tetracycline resistance markers, etc.) were elucidated (Dubert et al., 2015). In other report, about 63% of the isolated *V. parahaemolyticus* strains were resistant to ampicillin, cephalexin, or kanamycin (Bhattacharya et al., 2000). Hence, appearance of resistance to actinonin is a predictable consequence, and it is necessary to study the resistance mechanism of *V. anguillarum* against actinonin.

It was reported that mechanisms causing PDF inhibitor resistance involve (i) mutations in the target gene, (ii) bypassing of the formylation pathway, or (iii) eﬄux of PDF inhibitor (Duroc et al., 2009). Notably, we could amplify genes involved in translation initiation including *pdf*, *fmt*, and *glyA* but failed to get *folD* fragment from mutants, and then we confirmed a fragment deletion happened in the gene *folD*. Interestingly, similar mutations in the gene *fold* of *S. enterica* and *B. subtilis* had been described previously (Duroc et al., 2009). The loss of function of *folD* could inactivate translation initiation pathway that uses 10-formyl-THF, which led to a dramatic decrease of growth rate of Δ*folD* mutants. It is proposed that, in addition to *folD*, mutations in the genes involved in eﬄux pump, modification of actinonin or coding enzymes that degrade actinonin might also happened. Additionally, the RT-PCR results showed the expression of genes *pdf*, *atpH*, and *ahpF* were significantly regulated, which suggested that *V. anguillarum* mutants might develop an adaptation mechanism to survive in high concentration of actinonin.

Collectively, it is evident that VaPDF can be a good target for anti-*Vibrio* agents screening. And actinomycetes isolated from marine could be promising candidates for treating pathogens in marine aquaculture. It will also be very interesting to find more anti-*Vibrio* compounds with the present HTS model and develop the corresponding anti-bacteria drugs in the future.

## Author Contributions

NY and CS conceived and designed the experiments. NY performed all of the experiments. NY and CS analyzed the data, prepared the figures and wrote the paper. All authors reviewed the manuscript.

## Conflict of Interest Statement

The authors declare that the research was conducted in the absence of any commercial or financial relationships that could be construed as a potential conflict of interest.

## Footnotes

1 http://blast.ncbi.nlm.nih.gov/Blast.cgi

2 http://www.ncbi.nlm.nih.gov
