# Supplementary material for: The Inhibition and Resistance Mechanisms of Actinonin, Isolated from Marine Streptomyces sp. NHF165, against Vibrio anguillarum
Source: Front Microbiol. 2016 Sep 13;7:1467. doi: 10.3389/fmicb.2016.01467 (PMC5020083; doi:10.3389/fmicb.2016.01467)
Supplement: Supplementary file 1 [file Data_Sheet_1.DOCX]

**The inhibition and resistance mechanisms of actinonin, isolated from marine *Streptomyces* sp. NHF165, against *Vibrio anguillarum***

*Na Yang^1,2^, Chaomin Sun^1,2^**

*^1^Key Laboratory of Experimental Marine Biology, Institute of Oceanology, Chinese Academy of Sciences, Qingdao, 266071, China.*

*^2^Laboratory for Marine Biology and Biotechnology, Qingdao National Laboratory for Marine Science and Technology, Qingdao, 266071, China.*

*^*^ Corresponding author*

*Chaomin Sun Tel.: +86 532 82898857; fax: +86 532 82898857.*

*E-mail address:* [*sunchaomin@qdio.ac.cn*](mailto:sunchaomin@qdio.ac.cn)

**Supplementary Information**

**Table S1.** **Samples location in the South China Sea**.

| Sediment sample No. | Longitude (E) | Latitude (N) | Depth  (m) |
| --- | --- | --- | --- |
| 31 | 118°29.415′ | 11°58.721′ | 2488 |
| 32 | 117°57.276′ | 11°57.896′ | 2939 |
| 33 | 116°54.184′ | 12°16.136′ | 2974 |
| 37 | 116°28.749′ | 10°29.668′ | 1127 |
| 54 | 110°30.24′ | 14°33.112′ | 1039 |
| 65 | 109°53.171′ | 16°3.576′ | 880 |
| 69 | 109°59.417′ | 17°59.83′ | 98 |
| 76 | 111°45.43′ | 20°15.295′ | 79 |

**Table S2.** **Nucleotide sequences of the primers used for RT-PCR amplification.**

| Primer | Sequence (5’ to 3’) |
| --- | --- |
| ahpF-For | GTATTCTACCCGCTCTT |
| ahpF-Rev | GCTTTGATACCAGCAGCCCAC |
| atpH For | GCCGATTAAAGGCTCTT |
| atpH Rev | CGCTGCTCAAGTTTGGTGCT |
| fhuD For | TACAACCCGTTAGCCAGCCCAGAT |
| fhuD Rev | CCAACACCAGCAAACTAC |
| gtlB For | AACGAGCAAGGTCAAGTCACGG |
| gtlB Rev | GCCCATAGCAAGGAAAGCC |
| hisC For | CTTGTCAGCCATTCGTGC |
| hisC Rev | GCGGTCTTTAGTCATTTCCA |
| pdf For | GCAGCAGAAGTCACAG |
| pdf Rev | AATGGCGAAAGGTAAT |
| pdhA For | CCTTACATTCAAACGGTA |
| pdhA Rev | TTCACGGCTATCAGAACGA |
| rplL For | TACTAACGAGCAAATCCTAG |
| rplL Rev | GCTGGAGCGCCGTCAAC |
| srfAC For | CCCTGTTTGGGCAACGACC |
| srfAC Rev | CGCAAGGCTAAGCAGTTC |

For, forward; Rev, reverse.


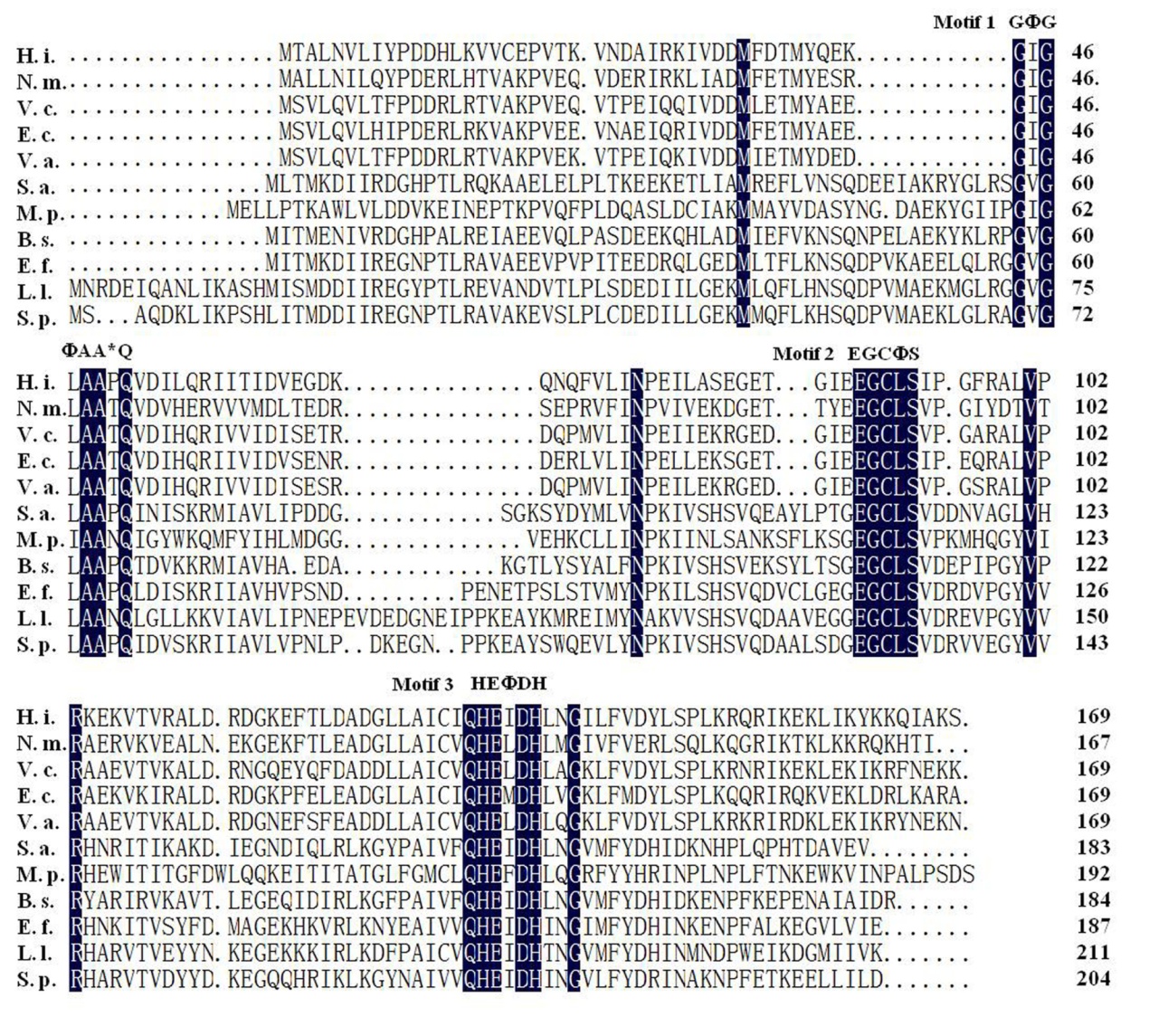


**Figure S1.** **Comparison of different peptide deformylase sequences based on amino acids**. Three conserved motifs across both Class Ⅰ and Class Ⅱ are highlighted in dark blue. Class Ⅰ: H. i., *Haemophilus influenzae* Rd (NP438782). N. m., *Neisseria* *meningitidis* MC58 (NC003112). V. c., *Vibrio* *cholerae* El tor N16961 (NP229705). E. c., *Escherichia* *coli* O157:H7 (BAB37575). V. a., *Vibrio* *anguillarum* YN (KU214433). Class Ⅱ: S. a., *Staphylococcus* *aureus* N315 (BAB42188). M. p., *Mycoplasma* *pneumoniae* M129 (NP109933). B. s., *Bacillus* *subtilis* (AIW29672). E. f., *Enterococcus* *faecalis* V583 (NP816678). L. l., *Lactococcus* *lactis* subsp. *lactis* Il1403 (NP266716). S. p., *Streptococcus* *pyogenes* M1 (AAK34651).


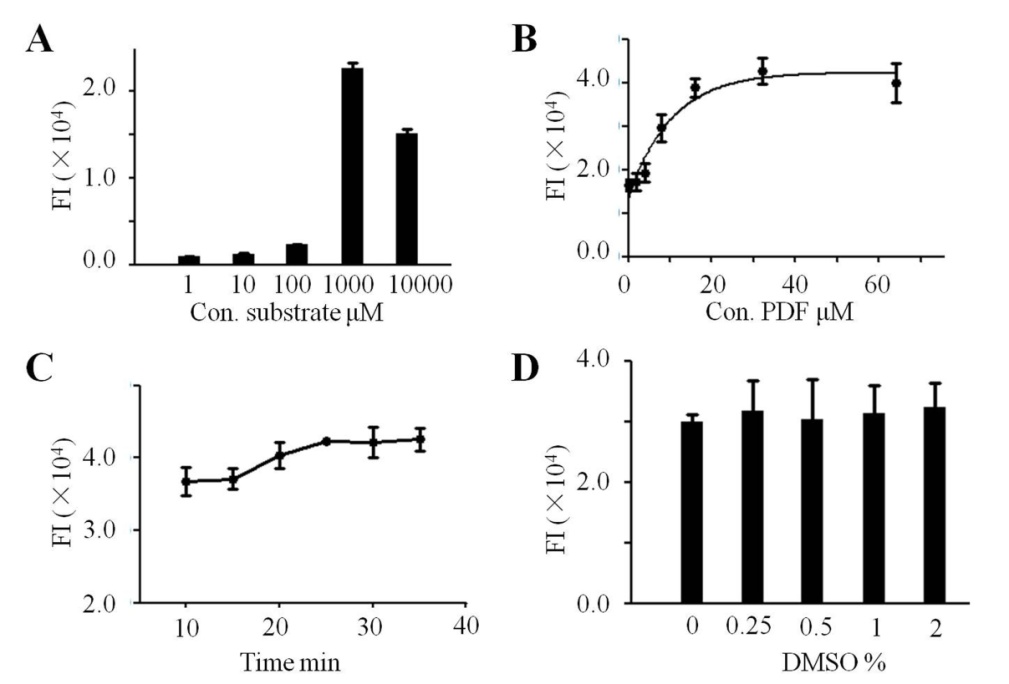


**Figure S2.** **Optimization of screening conditions**. (**A**) Optimization of formyl-Met-Ala-Ser. (**B**) Optimization of VaPDF concentration. (**C**) Optimization of reaction time. (**D**) DMSO concentration tolerance test.


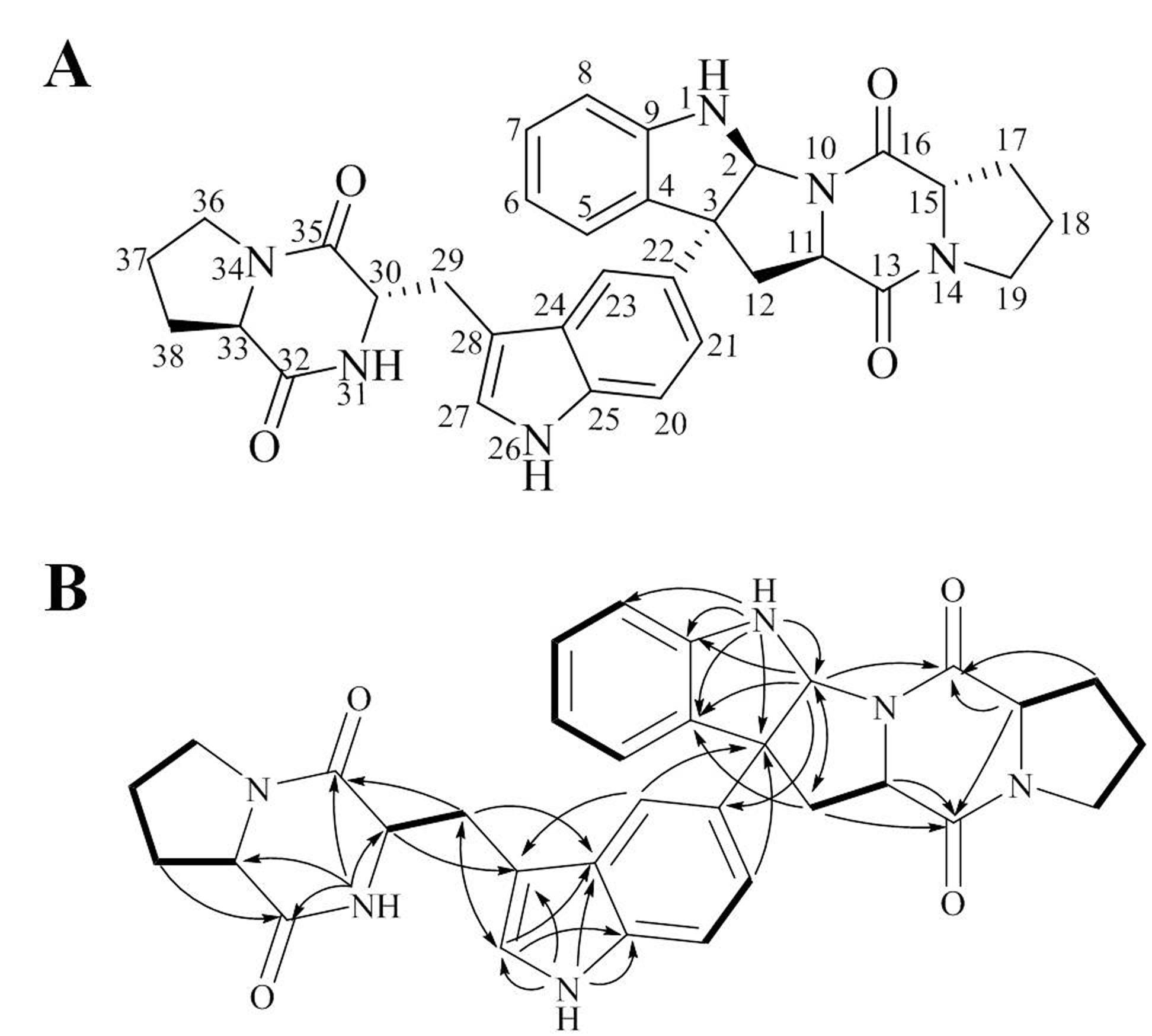


**Figure S3.** **Characterization of compound 2 isolated from strain *Streptomyces* sp. NHF165.** (**A**) The Structure of compound **2**. (**B**) The key ^1^H-^1^H COSY (bold line) and HMBC (H→C) correlations of compound **2.**

**.**


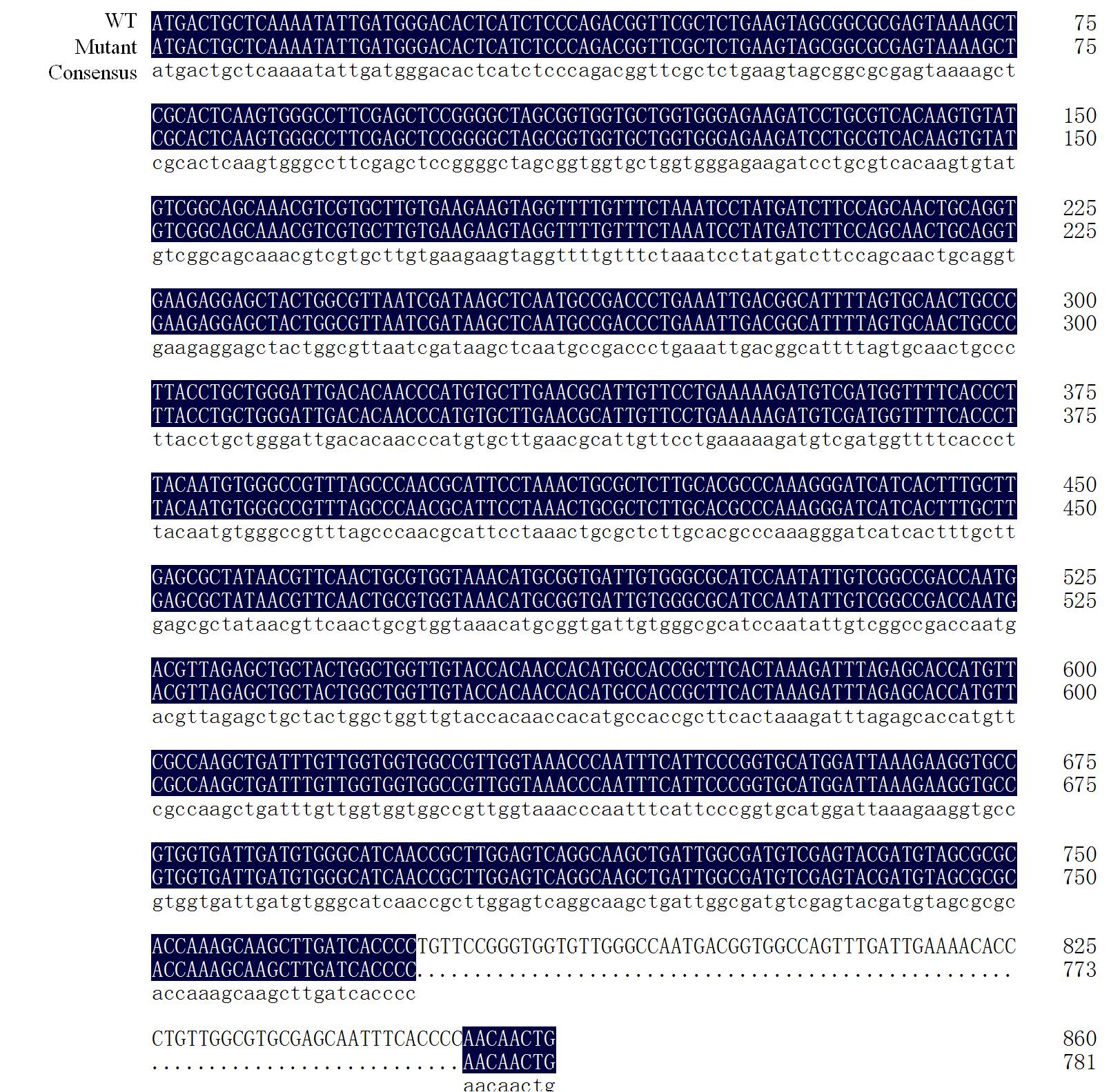


**Figure S4. Comparison of *folD* gene sequences between wild type and mutant *V. anguillarum* strains.** WT, wild type. Mutant, Δ*folD* mutant. Consensus, identical nucleotides.
